# Tissue plasminogen activator dose and pulmonary artery pressure reduction in catheter directed thrombolysis of submassive pulmonary embolism

**DOI:** 10.1371/journal.pone.0211701

**Published:** 2019-02-06

**Authors:** Darshan C. Patel, Ron C. Gaba, Li Liu, R. Peter Lokken

**Affiliations:** 1 Department of Radiology, University of Illinois Health, Chicago, Illinois, United States of America; 2 Department of Epidemiology and Biostatistics, School of Public Health, University of Illinois at Chicago, Chicago, Illinois, United States of America; 3 Department of Radiology and Biomedical Imaging, University of California, San Francisco, San Francisco, California, United States of America; Osaka University Graduate School of Medicine, JAPAN

## Abstract

**Purpose:**

The purpose of this study is to assess the incremental effect of tissue plasminogen activator (t-PA) dose on pulmonary artery pressure (PAP) and bleeding during catheter directed thrombolysis (CDT) of submassive pulmonary embolism (PE).

**Materials and methods:**

Records of 46 consecutive patients (25 men, 21 women, mean age 55±14 y) who underwent CDT for submassive PE between September 2009 and February 2017 were retrospectively reviewed. Mean t-PA rate was 0.7±0.3 mg/h. PAP was measured at baseline and daily until CDT termination. Mixed-effects regression modeling was performed of repeated PAP measures in individual patients. Bleeding events were classified by Global Utilization of Streptokinase and Tissue Plasminogen Activator for Occluded Coronary Arteries (GUSTO) and t-PA dose at onset.

**Results:**

Mean t-PA dose was 43.0±30.0 mg over 61.9± 28.8 h. Mean systolic PAP decreased from 51.7±15.5 mmHg at baseline to 35.6±12.7 mmHg at CDT termination (p<0.001). Mixed-effects regression revealed a linear decrease in systolic PAP over time (β = -0.37 (SE = 0.05), p<0.001) with reduction in mean systolic PAP to 44.8±1.9 mmHg at 12 mg t-PA/20 h, 39.5±2.0 mmHg at 24 mg t-PA/40 h, and 34.9±2.1 mmHg at 36 mg/60 h. No severe, one moderate, and 8 mild bleeding events occurred; bleeding onset was more frequent at ≤24 mg t-PA (p <0.001). One patient expired from cardiopulmonary arrest after 16 h of CDT (15.4 mg t-PA); no additional intra-procedural fatalities occurred.

**Conclusion:**

Increased total t-PA dose and CDT duration were associated with greater PAP reduction without increased bleeding events.

## Introduction

Prospective trials investigating catheter directed thrombolysis (CDT) of submassive pulmonary embolism (PE) demonstrated reduction of pulmonary artery pressure (PAP) [1] and right heart strain [2, 3] in CDT treatment arms. These results are corroborated by findings from the prospective multicenter Pulmonary Embolism Response to Fragmentation, Embolectomy, and Catheter Thrombolysis (PERFECT) registry [4] and retrospective studies [5–12]. However, thrombolytic dose and duration were limited to 4–24 mg tissue-plasminogen activator (t-PA) over 2–24 h in the prospective trials [1–3]; patients in PERFECT received a mean t-PA dose of 28±11 mg over 23.2±8.1 h using ultrasound assisted thrombolysis (USAT) or 20.8±11.5 h with standard CDT [4]. The ULTIMA study [2] reported a 10% minor bleeding complication rate and no major bleeding; the SEATTLE II study [1, 13] reported a 20%, 10.7% and 0.7% incidence of mild, moderate and severe bleeding events respectively. In the OPTALYSE PE study, four patients (4%) suffered 5 major bleeding events, included one intracranial hemorrhage attributed to CDT; this patient had been randomized to receive 12–24 mg t-PA over 6 h, a higher dose over a shorter duration compared to the ULTIMA (10–20 mg t-PA over 15 h) and SEATTLE II (24 mg t-PA over 12–24 h) trials [3]. The highest rates of major bleeding in these trials have occurred with t-PA dosage of ≥2 mg/h, raising the possibility that administration rates above 1 mg/h may decrease safety of CDT [14].

While these studies indicate that CDT reduces pulmonary hypertension and right heart strain in patients with submassive PE, the incremental impact of increased cumulative thrombolytic dose on PAP and hemorrhagic complications, particularly with cumulative t-PA doses above 24 mg, has not been established. We have therefore investigated the hypothesis that CDT with a low dose rate (≤1 mg/h) extended beyond published trial protocols further reduces PAP without an increase in bleeding complications.

## Materials and methods

This single-center retrospective cohort study was compliant with the Health Insurance Portability and Accountability Act. Institutional Review Board approval was obtained with waiver of informed consent for review of records.

### Study population

Medical records and imaging studies were reviewed for 46 consecutive patients (25 men, 21 women) who underwent CDT for submassive PE between September 2009 and February 2017 at a tertiary care hospital. Submassive PE was defined by 2011 American Heart Association (AHA) guidelines [15]. PE was confirmed with computed tomography angiography (CTA, n = 44), lung perfusion single photon emission computed tomography-computed tomography (SPECT-CT, n = 1), and digital subtraction angiography (DSA, n = 1). This study includes 17 patients who were previously published [16] in order to provide new information regarding changes in PAP in serial measurements during CDT. Upon review of data, the prior study included 2 patients who met AHA criteria for massive pulmonary embolism, including one patient who developed intracranial hemorrhage during thrombolysis; these patients were therefore excluded from this analysis.

### Baseline patient characteristics

Mean patient age was 55±14 years; 31 (67%) patients were African American, 6 (13%) Caucasian, and 6 (13%) Hispanic or Latino (Table 1). Mean time from symptom onset to PE diagnosis and initiation of CDT was 2.6±2.9 d and 3.4±3.2 d respectively. The most common presenting symptoms were dyspnea (n = 42, 91%), chest pain (n = 24, 52%), and syncope (n = 11, 24%). CT findings of right ventricle (RV) strain were present in 43 of 44 (98%) patients with a mean RV/left ventricle (LV) diameter ratio of 1.6±0.7. Echocardiographic evidence of right heart strain [15] was observed in 35 of 42 (83%) patients. Twenty-seven (59%) patients had an elevated simplified pulmonary embolism severity index (sPESI) score [17]. Three patients had a contraindication to systemic lysis (C3-C7 laminectomy 20 d prior, n = 1; gastric band placement 7 d prior, n = 1; uterine myomectomy 8 d prior, n = 1). CDT was the only thrombolytic therapy performed for all patients.

**Table 1:** Patient demographic and clinical characteristics (n = 46).

GENDER	FEMALE	21 (46%)
	Male	25 (54%)
Mean Age (years)		55±14
Body mass index (kg/m^2^)		34.5±10.0
Ethnicity/race	African American	31 (67%)
	Caucasian	6 (13%)
	Hispanic or Latino	6 (13%)
	Middle Eastern	3 (7%)
Risk factors	Obesity	29 (63%)
	Hypertension	23 (50%)
	Immobility within 30 d	11 (24%)
	Previous venous thromboembolism	9 (20%)
	Diabetes	7 (15%)
	Recent surgery (within 30 d)	6 (13%)
	Active malignancy	6 (13%)
	Oral contraceptive use	3 (7%)
	Hypercoagulable disorder	2 (4%)
	End stage renal disease	2 (4%)
	Family history of venous thromboembolism	1 (2%)

### CDT technique

The right (n = 41) or left (n = 2) common femoral vein, or right internal jugular vein (n = 2), was accessed with a 21-gauge needle (Micropuncture Introducer Set; Cook Medical, Bloomington, Indiana) under real-time sonographic guidance. Vascular access was dilated to accept a 5-French (n = 1), 7-French (n = 30), or 8-French (n = 15) vascular sheath (Pinnacle; Terumo, Somerset, New Jersey). A 7-French (APC, Cook Medical, Bloomington Indiana; n = 43) or 5-French (SOS Omni Flush, Angiodynamics, Latham New York; n = 1; APC, Cook Medical, Bloomington Indiana; n = 1) was positioned in the main PA and baseline PAP determined. Angiography was performed with iohexol (Omnipaque 300; GE Healthcare, Little Chalfont, United Kingdom). CDT was performed with a single catheter in the right or left PA (n = 45) or bilateral PA catheters (n = 1). When a single catheter was used, it was repositioned into the contralateral PA at operator discretion in 17 patients. Tissue plasminogen activator (t-PA, Activase; Genentech, San Francisco, California) was instilled through the PA catheter(s) at a mean rate of 0.7±0.3 mg/h (range, 0.4–1.1 mg/h). The dose rate was per operator discretion based upon clinical acuity and bleeding risk. Intravenous heparin was administered concurrently with goal partial thromboplastin time (PTT) of 60–80 s. Patients were monitored in the intensive care unit (ICU) during CDT. Serial fibrinogen levels were measured every 6 h (range 185–946 mg/dL). Angiography with PAP measurement was performed daily until CDT termination. CDT was terminated at operator discretion based on symptomatic improvement, reduction in PAP and clot burden. Hemostasis was achieved at the venotomy site with manual compression upon termination. Mechanical clot disruption techniques were not utilized.

### Measured outcomes

Serial systolic PAP measured during daily follow-up was tabulated. Duration of ICU and total hospital stay were also recorded. Technical success was defined as successful PA catheter placement and fibrinolytic agent delivery [18].

Procedural (during CDT) and postprocedural (within 30 d of CDT) adverse events were categorized according to the Society of Interventional Radiology classification [19]. Bleeding complications were additionally classified according to GUSTO [20] and by onset before or after infusion of 24 mg t-PA [1, 2, 4, 11].

Patients were followed until death or the last documented clinical encounter. Post-CDT right heart function was assessed with echocardiography in 26 (56%) patients at a mean 10.4±1.9 months after CDT and with contrast enhanced CT in 13 (28%) patients at a mean 17.4±18.8 months after CDT. Echocardiographic evidence of right heart strain was categorized as normal, mild, moderate, or severe using guidelines from the American Society of Echocardiography [21]. Imaging outcomes of patients with baseline and follow-up echocardiography were stratified by total infusion of less than or greater than 24 mg t-PA.

### Statistical analysis

Mixed-effects regression models were employed to examine changes in repeated measures of PAP in individual patients by increasing cumulative t-PA dose over time. Random effects were estimated to account for correlations between repeated measurements from the same individual. Likelihood ratio tests and fit statistics including Akaike information criterion (AIC) and Bayesian information criterion (BIC) were used to select the best-fitted number of random effects, as well as variance-covariance structure of repeated measurements. Dose values at each time point were treated as time-varying covariates, and the interaction of dose with time was tested to examine how dose values affect PAP over time. Model estimates and standard errors were reported from the final model. Baseline vs. post CDT findings of right heart strain on echocardiography, severity of right heart strain on echocardiography and severity of right heart strain on CT were statistically compared using McNemar’s test, Wilcoxon Signed-Rank test, and T-test respectively. Distributions of mean cumulative t-PA dose and systolic PAP for patients who underwent echocardiography or CT follow-up were compared with the remaining cohort using the Mann-Whitney U test. Incidence of bleeding event onset before or after administration of 24 mg t-PA was compared using Fisher’s Exact Test. Analyses were performed with SAS (version 9.4, SAS Institute Inc., Cary North Carolina).

## Results

### Procedural characteristics

**Fig 1:**
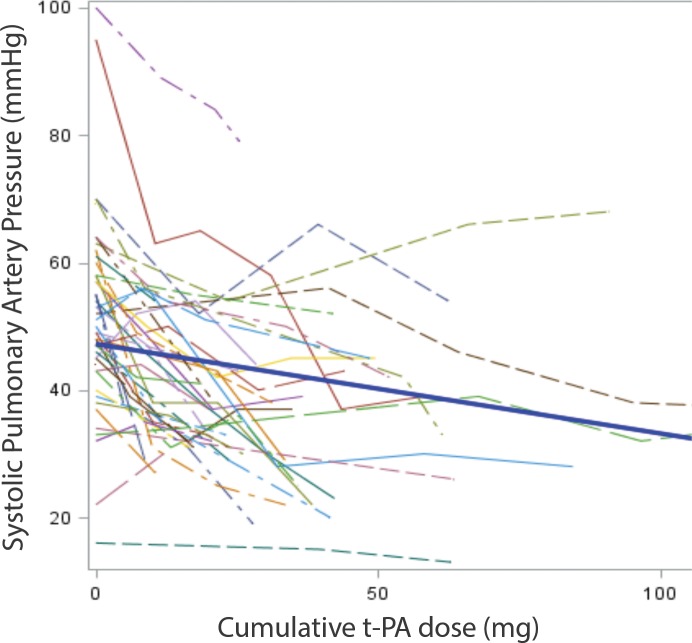
Systolic PAP as a function of cumulative t-PA dose for each patient (light lines). Mixed-effects regression model (dense line) revealed a significant linear decrease in systolic PAP (β = -0.37 (SE = 0.05), p < 0.001).

All procedures were technically successful. CDT was performed for a mean 61.9±28.8 h with mean cumulative t-PA dose of 43.0±30.0 mg. Intraprocedural angiography and PAP measurement occurred at a mean 19.5±4.5 (n = 46), 43.0±4.9 (n = 40), 63.9±5.7 (n = 26), 89.2±3.7 (n = 13), 113.5±2.1 (n = 4) and 138.0 h (n = 1) after initiation of CDT with corresponding mean cumulative t-PA dose of 11.8±4.8, 27.6±10.4, 42.4±14.4, 64.0±23.8, 98.9±31.3 and 158.3 mg respectively. Measured mean systolic PAP at these intervals was 43.0±12.3, 41.6±13.6, 37.6±14.5, 34.5±14.3, 48.0±17.3, and 39.0 mmHg. Mixed-effects regression revealed a linear decrease in systolic PAP by increasing t-PA dose (Fig 1), with a 0.3 mmHg/h decrease in systolic PAP (β = -0.37 (SE = 0.05), p < 0.001), and no observed association between mean patient dose and this trend (p = 0.37). Recorded and mixed-effects regression fitted systolic PAP by t-PA dose and time are summarized in Table 2.

**Table 2:** Mean systolic pulmonary artery pressure and cumulative t-PA dose at follow-up intervals.

RECORDED*			MIXED-EFFECTS REGRESSION MODEL VALUES*		
Mean follow-up interval (hours)	Mean cumulative administered t-PA dose (mg)	Mean systolic pulmonary artery pressure (mmHg)±SD	Follow-up interval (hours)	Cumulative administered t-PA dose (mg)	Mean systolic pulmonary artery pressure (mmHg)±SE
Baseline (n = 46)	N/A	51.7±15.5	Baseline	N/A	50.8±2.1
19.5±4.5 (n = 46)	11.8±4.8	43.0±12.3	20	12.0	44.8±1.9
43.0±4.9 (n = 40)	27.6±10.4	41.6±13.6	40	24.0	39.5±2.0
63.9±5.7 (n = 26)	42.4±14.4	37.6±14.5	60	36.0	34.9±2.1

*All p values < 0.001.

### Evaluation of right heart function after CDT

**Fig 2:**
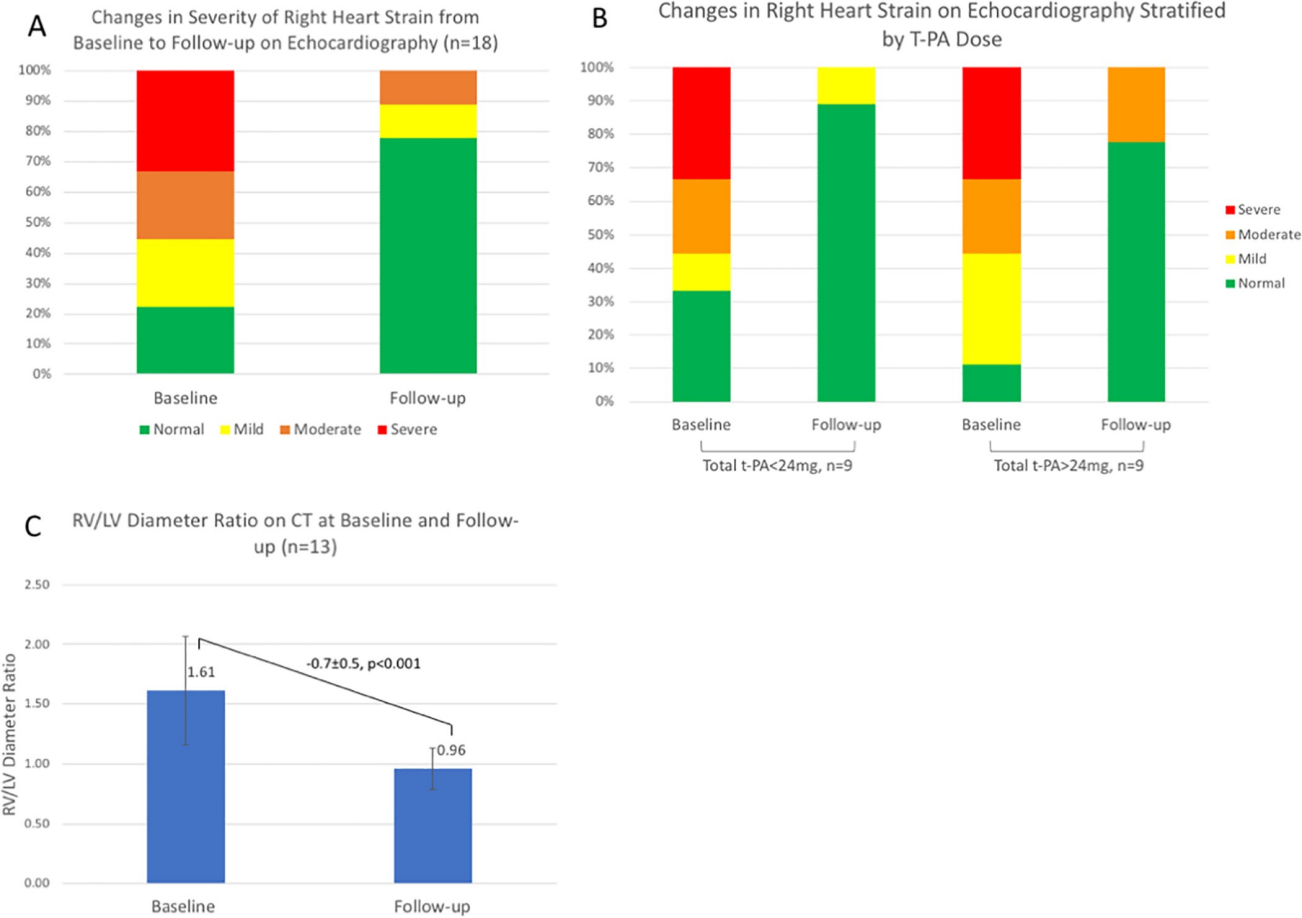
(A) Changes in severity of right heart strain on echocardiography from baseline to follow-up (p<0.001). (B) Changes in right heart strain on echocardiography stratified by total t-PA dose. (C) Decrease in RV/LV diameter ratio on CT from baseline to follow-up (p<0.001).

Patients were followed for a mean 22.9±26.7 months. Both baseline and follow-up echocardiograms were performed in 18 (39%) patients at a mean follow-up of 10.4±19.6 months post CDT. Among patients with both baseline and follow-up echocardiograms, right heart strain was present in 4 of 18 (22%) patients on follow-up compared to 14 of 18 (78%) at baseline (p = 0.002); severity of right heart strain also decreased between baseline and follow-up (Fig 2A, p<0.001). Fig 2B illustrates changes in severity of right heart strain on echocardiography stratified by t-PA dose. Among patients with follow-up echocardiography, 8 of 9 (88.9%) patients who received >24 mg t-PA had echocardiographic evidence of right heart strain at baseline compared to 6 of 9 (66.7%) of patients who received <14 mg t-PA. On follow-up echocardiography, 7 of 9 (77.8%) patients who received >24 mg t-PA had no evidence of right heart strain, compared to 8 of 9 (88.9%) patients who received <24 mg t-PA. Patients with both baseline and follow-up echocardiograms were administered a similar cumulative t-PA dose (35.1±23.9 mg, p = 0.17) and had similar systolic PAP at baseline (49.5±12.8 mmHg, p = 0.68) and CDT completion (35.1±14.8 mmHg, p = 0.55) compared to the remainder of the cohort.

Baseline and follow-up contrast enhanced CT was performed in 13 (28%) patients at a mean 17.4±18.8 months post CDT and demonstrated a mean RV/LV diameter ratio decrease from 1.6±0.7 to 1.0±0.2 (Fig 2B, p<0.001). Patients with CT follow-up received a similar dose of t-PA (39.9±23.4 mg, p = 0.59) and had similar PAP at baseline (47.8±15.6 mmHg, p = 0.65) and CDT completion (32.5±13.4 mmHg, p = 0.35) compared to patients without CT follow-up.

### Clinical outcomes and adverse events

Presenting symptoms resolved in 45 (98%) of patients with a mean time to resolution of 1.8±0.9 d. Mean ICU stay was 4.9±3.1 d and mean total hospital stay was 8.0±5.4 d.

An intra-procedural complication occurred in 9 (19.6%) patients (Table 3). One (2%) patient died 16 h after initiation of CDT (15.4 mg t-PA) from cardiopulmonary arrest attributed to PE (SIR grade 5). No additional moderate or severe intra-procedural adverse events occurred. Mild intra-procedural bleeding (SIR grade 1, GUSTO grade 1) occurred in 8 patients (18%) at a mean 38.4±20.4 h after initiation of CDT; mild bleeding complications included small volume hemoptysis (n = 2), small hematoma or oozing at a venous access site (n = 5), and transient gross hematuria after Foley catheter insertion during CDT (n = 1). No moderate or severe intra-procedural bleeding events occurred.

**Table 3:** Safety outcomes (n = 46).

MEAN ICU LENGTH OF STAY (D)	4.9±3.1	
Mean total hospital length of stay (d)	8.0±5.4	
All cause 30-day mortally, n (%)	2 (4%)	
All procedural complications	**GUSTO mild, n = 8 (17%)**	
	Occurrence at ≤24 mg t-PA infused, n (%)	7 (88%)*
	Occurrence at >24 mg t-PA infused, n (%)	1 (12%)
	**SIR grade 5 (death), n = 1 (2%)**	
	Occurrence at ≤24 mg t-PA, n (%)	1 (100%)
	Occurrence at >24 mg t-PA, n (%)	0 (0%)
All post-procedural complications (within 30 d of CDT)	**GUSTO moderate, n = 1 (2%)**	
	Cumulative t-PA dose administered ≤24 mg, n (%)	1 (100%)
	Cumulative t-PA dose administered >24 mg, n (%)	0 (0%)
	**SIR grade 5 (death), n = 1 (2%)**	
	Cumulative t-PA dose administered ≤24 mg, n (%)	0 (0%)
	Cumulative t-PA dose administered >24 mg, n (%)	1 (100%)

Intensive care unit (ICU), catheter directed thrombolysis (CDT), Global Utilization of Streptokinase and Tissue Plasminogen Activator for Occluded Coronary Arteries (GUSTO), Society of Interventional Radiology (SIR).

*p<0.001

Post-procedural complications were observed in 2 patients. A patient who underwent CDT for 21.5 h (10.5 mg t-PA) developed retroperitoneal bleeding 24 hours after CDT termination, requiring transfusion with 1 unit packed red blood cells (SIR grade 2, GUSTO grade 2), IVC filter placement, and cessation of systemic anticoagulation for 10 d; the patient recovered and the IVC filter was retrieved 136 d later. One patient died of sepsis 22 d after completion of CDT (SIR grade 5). No additional post-procedural deaths or bleeding events were observed within 30 d of CDT.

Of the 9 total bleeding complications (1 moderate, 8 mild), 8 (89%) occurred at a cumulative t-PA infusion dose of ≤24 mg (Table 3).

## Discussion

Meta-analyses of prospective clinical trials evaluating systemic fibrinolysis for submassive PE have suggested a decrease in all-cause mortality [22] or clinical deterioration [23, 24] compared to anticoagulation alone, at the expense of an increased risk of major bleeding. Direct instillation of a lower dose of fibrinolytic agent into the PA via CDT has been investigated with the goal of achieving therapeutic benefit while minimizing bleeding complications. However, the optimal dose, duration, and technique of CDT is not established. The higher rates of major bleeding in the SEATTLE II study, in which the majority of patients received 2 mg t-PA/h, and in the OPTALYSE PE trial arm receiving 12–24 mg/t-PA over 6 h, suggest that a dose rate of over 1 mg t-PA/h may result in greater hemorrhagic complications [1, 3, 14]. The majority of prospective and retrospective studies have assessed CDT with ≤24 mg t-PA; [1–5, 7–9, 11] the impact of t-PA dose >24 mg, administered at a low (≤1 mg/h) dose rate, upon PAP reduction and bleeding risk is unclear.

**Fig 3:**
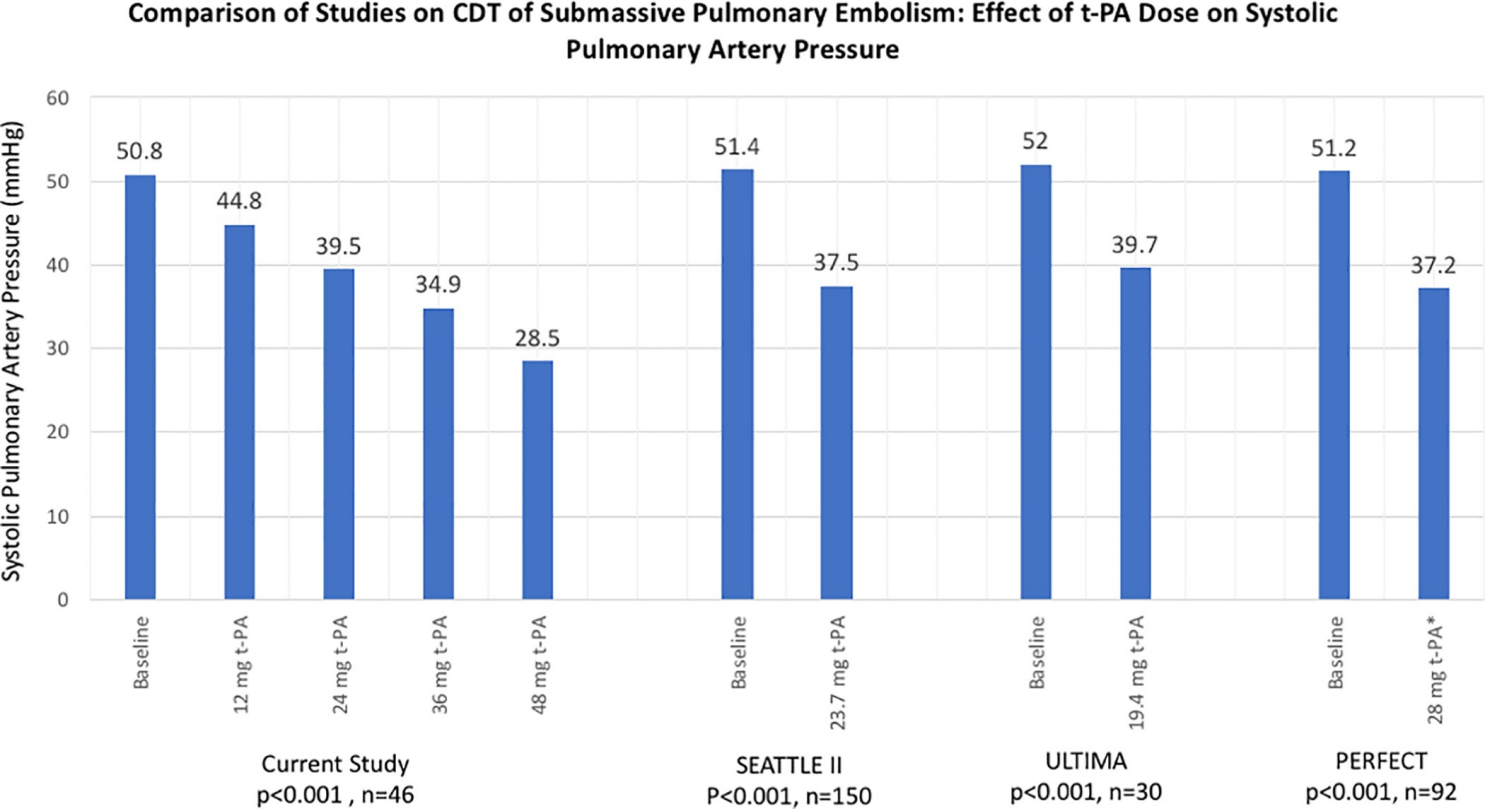
Comparison of t-PA dose effect on systolic pulmonary artery pressure in prior CDT studies for submassive pulmonary embolism. * 28 mg t-PA or 2,67,101 International units of urokinase.

In this study, a decrease in mean systolic PAP from 50.8±2.1 to 39.5±2.0 mmHg (11.3 mmHg reduction) was observed after CDT with 24 mg t-PA administered at a mean rate of 0.7 mg/h; this reduction is similar to the prospective ULTIMA (12.3 mm Hg reduction) [2] and SEATTLE II (14.5 mm Hg reduction) [1] trials and initial results of the PERFECT registry (14.0 mmHg reduction) [4]. However, the results of this study suggest that extending the duration of thrombolysis to administer >24 mg t-PA further decreases systolic PAP: additional reductions of 4.6 mmHg and 6.4 mmHg were observed with increased cumulative t-PA dose from 24 to 36 mg and 36 to 48 mg respectively (Fig 3). Extrapolation of the observed linear decrease in systolic PAP for the higher t-PA doses administered to this cohort should be performed with caution, as the number of patients who received, for example, >48 mg (n = 12), or underwent CDT for >72 h (n = 13), may be inadequate to derive reliable conclusions.

Our findings raise the possibility that extending the duration of CDT to administer over 24 mg t-PA may further reduce prevalence of right heart strain in follow-up imaging. Although a minority of patients in our study underwent echocardiography or CT follow-up, these patients had similar distributions of cumulative t-PA dose and systolic PAP compared to patients without imaging follow-up and appear representative of the entire cohort. In the ULTIMA [2] and SEATTLE II [1] studies a 0.35±0.22 and 0.42±0.36 improvement in RV/LV diameter ratio was observed, compared to 0.65±0.52 in this study; however, the greater mean improvement in this study may be attributable to the long interval between CDT termination and echocardiography (10.4 mo) and CT (17.4 mo). As illustrated in Fig 2B, among the subset of patients with baseline and follow-up echocardiography (n = 18), patients who received >24 mg t-PA were not more likely to have normal echocardiography on follow-up. However, a higher proportion of these patients also had abnormal baseline echocardiograms compared to patients who received <24 mg t-PA, and may have been selected for more prolonged CDT at operator discretion due to greater PE severity and lack of response to CDT at <24 mg t-PA. The impact of extending CDT on improvement of right heart function would require confirmation in further studies.

An increase in bleeding events was not observed with higher cumulative thrombolytic dose and duration of CDT. A total of 8 (17%), 1 (2%), and 0 (0%) GUSTO-defined minor, moderate and severe bleeding complications occurred; bleeding events were observed more commonly with ≤24 mg t-PA administered (Table 3). This statistical observation should be interpreted with caution, as prior studies have demonstrated increased bleeding with more prolonged thrombolysis [25]; in addition, two major hemorrhagic events, including one intracranial hemorrhage, occurred in 18 patients assigned to the highest dose arm (12–24 mg t-PA over 6 h) in the OPTALYSE PE study[3]. However, the results of the presented cohort suggest that prolongation of CDT with a low dose rate (<1 mg t-PA/h) beyond 24 mg t-PA to the average dose of 43.0±30.0 mg administered in this study was well tolerated. We observed fewer moderate and severe bleeding events than in the SEATTLE II study, and a similar low incidence of clinically significant bleeding events compared to the PERFECT and ULTIMA studies (Table 4).

**Table 4:** Comparison of bleeding complications to prior studies.

	Study			
Bleeding Complication Severity	Current Study.^1^ (n = 46)	ULTIMA (n = 30)	SEATTLE II^1^ (n = 150)	PERFECT^2^ (n = 101)
**Mild, n (%)**	8 (17.4%)	3 (10.0%)^3^	(20%)	13 (12.8%)
**Moderate, n (%)**	1 (2.2%)	N/A	16 (10.7%)	N/A
**Major, n (%)**	0 (0%)	0 (0%)^4^	1 (0.7%)	0 (0%)

^1^Bleeding complication categorized by Global Utilization of Streptokinase and Tissue Plasminogen Activator for Occluded Coronary Arteries (GUSTO)

^2^Bleeding complications categorized by Society of Interventional Radiology Practice Guidelines

^3^Clinically overt bleeding not fulfilling criteria of major bleeding

^4^Bleeding associated with a fall in hemoglobin ≥2.0 g/dL or with transfusion of ≥2 units of red blood cells or involvement of a critical site (intracranial, intraspinal, intraocular, retroperitoneal, intra-articular or pericardial, or intramuscular with compartment syndrome).

Although extending thrombolysis to an average 43.0±30.0 mg t-PA was associated with an additional decrease in PAP compared to 24 mg in this cohort, the benefit of further PAP reduction on clinical outcomes such as exercise tolerance was not assessed and remains unestablished. Any clinical benefit of extended CDT must be balanced with potentially increased resource utilization in the form of prolongation of ICU length of stay during CDT. Although increased CDT duration likely lengthened ICU stay (mean 4.9±3.1 d), the average hospital stay (8.0±5.4 d) was similar to 8.9±3.4 d in ULTIMA [2], 8.8±5 d in SEATTLE II [1], and 8.2±4.8 d in PERFECT [4].

Comparisons of results in this study of CDT are made to prior studies using USAT [1–4]. Based upon current knowledge, the comparison is valid, as a benefit of USAT over CDT has not been demonstrated. Retrospective studies by Liang et al. and Graif et al. reported no difference in hemodynamic outcomes or procedural complication rates between USAT and standard CDT using conventional straight or pigtail-shaped multi-sidehole catheters [12, 26]. Within the PERFECT registry, no difference was observed in hemodynamic outcomes between USAT and CDT groups [4]. Similarly, the prospective, randomized BERNUTIFUL trial also showed no benefit to USAT compared to CDT for iliofemoral DVT [27]. Additional technical differences in the presented cohort study may have impacted rates of PA pressure reduction aside from t-PA dose. The majority of patients (97.8%) underwent CDT with a single pigtail catheter positioned ipsilateral to the greatest clot burden. In contrast, bilateral USAT catheters were utilized in 86% of patients in SEATTLE II and 87% in ULTIMA [1, 2]. Similar PA pressure reductions by t-PA dose were observed in this study compared to other studies (Fig 3). To our knowledge, no direct comparison of unilateral versus bilateral catheters has been performed to demonstrate the superiority of either approach, but it is conceivable that that bilateral PA catheters may result in differences in PA pressure reduction or bleeding complications compared to unilateral catheter placement. Finally, the rate of t-PA instillation in this study (mean 0.7 mg/h) is lower compared to 1.3 mg/h for 87% of patients in ULTIMA, 2 mg/h for 86% of patients in SEATTLE II, and up to 4 mg/h in OPTALYSE PE [1–3]. While higher dose rates may hasten hemodynamic improvement, the results of SEATTLE II and the highest dose-rate arm of OPTALYSE PE raise the possibility that higher infusion rates are also associated with more bleeding events [14].

The results of this study should be interpreted in the context of its non-randomized, retrospective study design. Without a control arm, it is unclear to what degree the incremental PAP reduction was attributable to additional t-PA, or from anticoagulation and time. However, in SEATTLE II [1] there was no discernible change in systolic PAP on echocardiography 48 h after CDT completion when compared to systolic PAP at CDT completion. This study is also limited by small sample sizes for t-PA doses above 48 mg t-PA (n = 12); therefore, mixed-effects regression modeling for doses above 48 mg should be extrapolated with caution. Detection of adverse events was dependent upon medical records, and minor complications in particular may be underreported. As with other studies of CDT for PE, assessment and follow-up for clinically important outcomes such as chronic thromboembolic pulmonary hypertension, exercise tolerance, and quality of life was not performed. Evaluation of RV function after CDT was not performed in a standardized fashion, and although the results of this study are promising, comparison of these results with prospective studies should be interpreted with caution. This study included a high proportion of African American patients (67%) compared to other studies (e.g. 14.7% in SEATTLE II, 3% in PERFECT, 36.6% in OPTALYSE PE) [1, 3, 4]; while representation of African American patients begins to fill a major deficit in the literature, patient demographics may theoretically affect external validity of this study. Finally, as with other studies on CDT for submassive PE, t-PA dose was not adjusted for body weight; weight-based standardization of t-PA dose in CDT may provide more rigorous pharmacokinetic data in future studies.

In conclusion, continuation of CDT beyond currently published trial protocols was associated with greater PAP reduction in patients with submassive PE without increased incidence of bleeding events. Although the clinical impact of accelerated PAP reduction by prolongation of CDT requires further investigation, an extended duration of thrombolysis administered at a low dose rate (<1 mg/h t-PA) was well tolerated and should be considered for future clinical trials assessing long-term outcomes of CDT for submassive PE.

## Supporting information

S1 Dataset Deidentified data. (XLSX)
